# Associations between FDG-PET and Ki 67-index in head and neck cancer: A meta-analysis

**DOI:** 10.1097/MD.0000000000017472

**Published:** 2019-10-04

**Authors:** Hans-Jonas Meyer, Peter Gundermann, Alexey Surov

**Affiliations:** a Department of Diagnostic and Interventional Radiology, University of Leipzig, Leipzig; b Department of Diagnostic and Interventional Radiology, University of Ulm, Ulm, Germany, Germany.

**Keywords:** FDG PET, HNSCC, Ki67, SUV

## Abstract

**Background:**

FDG-PET might be able to reflect histopathology features of tumors. Ki 67 in head and neck carcinomas (HNSCC). The present study sought to elucidate the association between Ki 67 index and SUV_max_ based upon a large patient sample.

**Methods:**

PubMed database was screened for studies analyzed the relationship between Ki 67 and SUV in HNSCC. Nine studies comprising 211 patients were suitable for analysis.

**Results:**

SUV_max_ increased with tumor grade and was statistically significant different between G1, G2, and G3 tumors. The ROC analysis for discrimination between G1/G2 and G3 tumors revealed an area under curve of 0.71. In the overall patient sample, SUV_max_ correlated statistically significant with Ki 67 index (r = 0.154, *P* = .032).

**Conclusion:**

The present study identified a weak correlation between SUV values and proliferation index Ki 67 index in HNSCC in a large patient sample. Therefore, SUV_max_ cannot be used as surrogate parameter for proliferation activity in HNSCC.

## 1 Introduction

Head and neck squamous cell carcinoma (HNSCC) is one of the most frequent malignancies in man with a rising incidence.^[1]^ Imaging plays a key role in correct diagnosis and tumor staging.^[2]^ The standard conventional imaging modalities for evaluating patients with HNSCC are computed tomography (CT) and magnetic resonance imaging (MRI). Furthermore, Fluorodeoxyglucose-Positron-emission tomography (FDG-PET) is increasingly used in clinical routine to provide information regarding tumor glucose-metabolism.^[2]^

PET can be quantified with Standardized Uptake value (SUV). It has been shown that SUV_max_ is strongly related to advanced stage, lymph node involvement, local extension, and tumor differentiation.^[3–6]^ Presumably, these associations might be caused by the ability of SUV to reflect histopathology in HNSCC, which was already shown in some studies.^[7–9]^ So, it has been shown that FDG uptake is strongly influenced by the expression of Glucose Transporter (GLUT)-proteins, a membrane-protein family, which mediates the glucose intake of cells.^[10]^ This is of special interest because GLUT expression is an independent prognostic marker to predict poor survival in various types of cancers.^[11]^ Furthermore, as reported previously, FDG-PET was associated with several histopathological parameters. So p16 positive carcinomas showed significantly lower SUV values than p16 negative tumors.^[12,13]^ Moreover, SUV_max_ can predict cell density in HNSCC.^[8]^ Additionally, SUV_max_ also correlated with Bcl2, a protein related with the cell cycle.^[13]^

One of the clinical important histopathological parameter is Ki 67, a widely used proliferation index, which is of prognostic relevance in various tumor entities.^[14]^ In HNSCC, it was shown that high Ki 67 expression was associated with overall poor prognosis, higher rate of lymph node metastasis.^[15,16]^

Thus, predicting Ki 67-index by imaging might be of special interest, which has been investigated by several studies in recent days using different imaging modalities.^[8,17]^

Presumably, PET parameters may well reflect proliferation activity. However, a recent meta-analysis comprising 3242 patients with various tumor entities identified only a moderate correlation coefficient of r = 0.44 between SUV_max_ and expression of Ki 67.^[18]^ Moreover, in HNSCC, the results of the published studies are very inconclusive. So Jacob et al, observed a strong correlation between SUV and Ki 67 (r = 0.83).^[19]^ However, other authors did not find significant correlations between PET and Ki 67.^[8,9]^ Furthermore, the reported data are based only on small number of patients/tumors.

Therefore, the purpose of the present study was to analyze associations between SUV and Ki 67-index in HNSCC in a large patient sample.

## 2 Methods

### 2.1 Data acquisition

On the first step, PubMed database was screened for studies analyzed the relationship between Ki67 and SUV in HNSCC: The search terms were Ki 67 OR Ki67 OR Ki-67 and SUV OR PET and HNSCC OR head and neck cancer. Overall, 140 items were collected. The 128 articles were excluded due to non-relation of HNSCC. Secondly, the full texts of the remaining 12 items were checked. After thorough analysis, 9 studies with 211 patients (Table 1) were included into the analysis.^[12,19–26]^

**Table 1:**
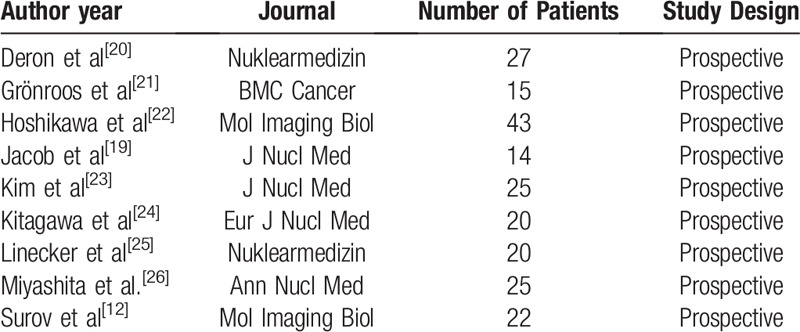
Overview about the included papers.

In 6 studies (112 patients) a PET scanner was used (66.7% of studies) and in 3 studies (74 patients) a PET-CT scanner was used (33.3%).

### 2.2 Statistical analysis

For statistical analysis Graph Pad Prism (GraphPad Software, La Jolla, CA) was used. Collected data were evaluated by means of descriptive statistics (absolute and relative frequencies). Categorical variables were expressed as percentages. *P* values < .05 were taken to indicate statistical significance in all instances. Spearman correlation coefficient was used to analyze the associations between SUV and Ki 67. Mann-Whitney *U* test was used for group comparisons. Finally, ROC-analysis was performed for discrimination of well/moderate differentiated tumors from poor differentiated tumors.

## 3 Results

### 3.1 SUV_max_ and tumor grade

**Figure 1:**
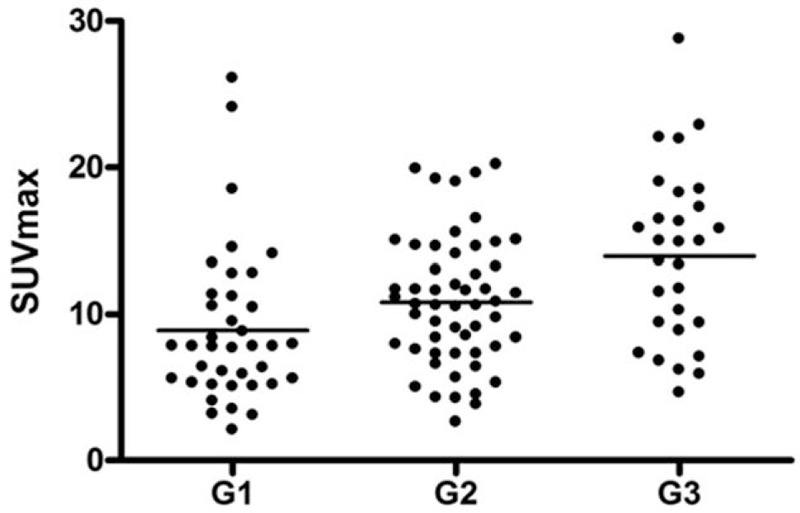
Scatter dot plot displaying the SUV_max_ values according to the tumor grading. There are statistically significant differences between tumor groups. Mean SUV_max_ 8.31 ± 4.6 for G1, 10.8 ± 4.3 for G2 and 13.8 ± 5.6, *P* = .004 for G1 vs G2 and *P* = .001 for G2 vs G3.

SUV_max_ increased with tumor grade and was statistically significant higher in G3 tumors in comparison to G2 lesions as well in comparison to G1 tumors (*P* < .0001) (Table 2). G2 tumors showed also higher SUV_max_ compared to G1 lesions (*P* = .004) (Fig. 1).

**Table 2:**
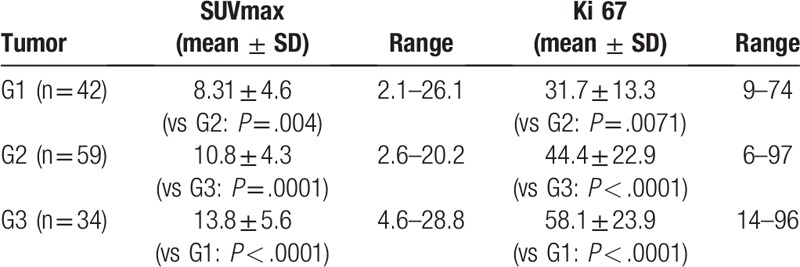
Overview about the tumors divided by grading.

**Figure 2:**
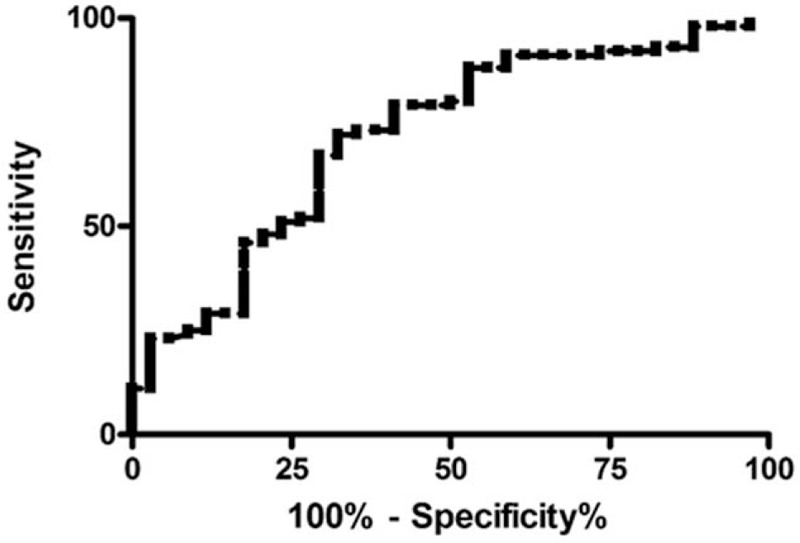
ROC analysis for discrimination between good/moderate differentiated tumors and poor differentiated tumors. The area under curve is 0.71 ± 0.05 (95% CI 0.61–0.82). With a cut off SUV_max_ value of 11.72, a sensitivity of 72% and specificity of 67.6%.

The ROC analysis for discrimination between G1/G2 and G3 tumors based on SUV_max_ values revealed an area under curve of 0.71 ± 0.05 (95% CI 0.61–0.82) (Fig. 2). A cut off SUV_max_ value of 11.72 resulted in a sensitivity of 72% and specificity of 67.6%.

### 3.2 Correlation between SUV_max_ and proliferation index Ki 67.

Ki 67-index increased significantly with tumor grades (*P* = .0071 for G1 vs G2 group und *P* < .0001 for G2 vs G3) (Table 2).

**Figure 3:**
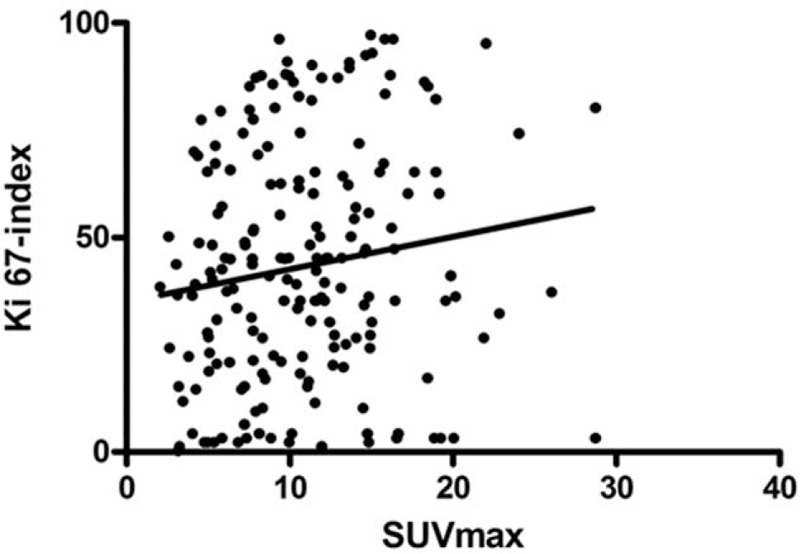
The correlation graph between SUV_max_ and Ki 67-index in the overall patient sample. The Spearman correlation coefficient is r = 0.154, *P* = .032.

**Figure 4:**
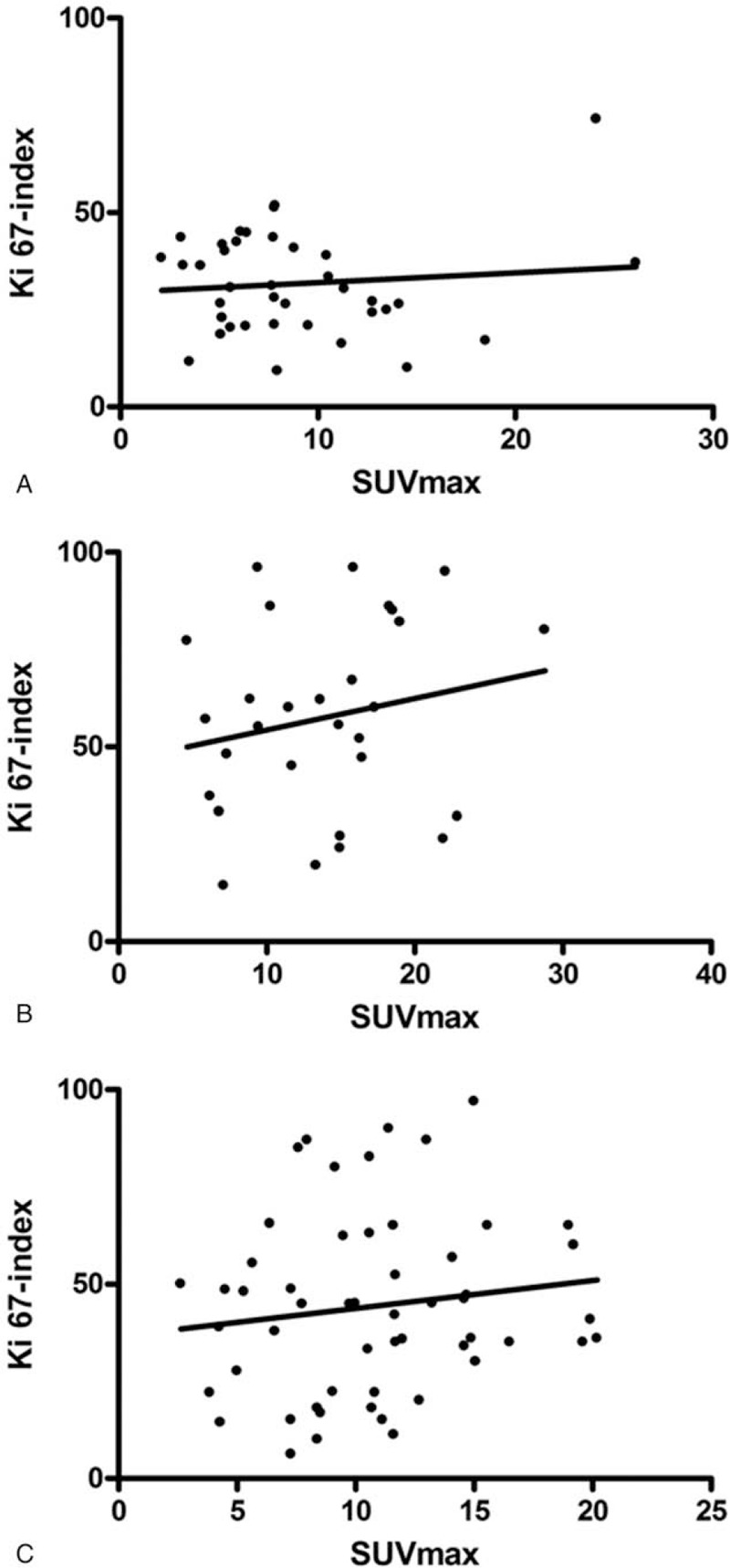
A. The correlation graph between SUV_max_ and Ki 67-index for good differentiated tumors (G1). The Spearman correlation coefficient is r = −0.146, *P* = .38. B. The correlation graph between SUV_max_ and Ki 67-index for moderate differentiated tumors (G2). The Spearman correlation coefficient is r = 0.125, *P* = .367. C. The correlation graph between SUV_max_ and Ki 67-index for poor differentiated tumors (G3). The Spearman correlation coefficient is r = 0.189, *P* = .326.

In the overall patient sample, SUV_max_ correlated statistically significant with Ki 67 (r = 0.154, *P* = .032) (Fig. 3). Divided into groups according to their tumor grades, the correlation coefficients were r = −0.146, *P* = .38 for well differentiated (G1), r = 0.125, *P* = .367 for moderately differentiated (G2) and r = 0.189, *P* = .326 for poorly differentiated (G3) tumors (Fig. 4A–C).

## 4 Discussion

The present analysis elucidated possible associations between SUV values derived from FDG-PET and Ki 67 index in HNSCC based on a large sample.

Ki 67 is a protein expressed in all phases of the cell cycle, except the G0-phase and can, therefore, estimate the fraction of proliferative cells in tissues.^[14]^ So, it has been shown that this proliferation index Ki 67 is an important characteristic of HNSCC. So, a higher expression of Ki 67 might indicate a poorer prognosis of the patients.^[15,16]^ Furthermore, it is associated with a higher rate of lymph node metastasis.^[15,16]^ Thus, the prediction of this histopathology parameter by imaging might be important in clinical routine.

The principle hypothesis why tumor proliferation and glucose metabolism displayed by FDG-PET are linked to each other is that tumor cell proliferation mainly depends on glycolysis for energy.^[18]^ In fact, in a large meta-analysis including various tumor entities could identify a moderate correlation of r = 0.44, which corroborates this hypothesis.^[18]^

Previously, only few studies investigated possible associations between SUV and Ki 67-index in HNSCC.^[7,8,12,18–26]^ As mentioned above, the reported data are inconclusive.^[18]^ Most authors could only identify a weak correlation between SUV and Ki 67.^[8,20,25]^ However, in the study of Hoshikawa et al no statistically significant correlation was observed.^[22]^ Contrary, a strong positive correlation was found by Jacob et al.^[19]^ The studies analyzed overall only a small number of patients. Moreover, the identified discrepancies of the studies might be caused by different tumor localizations included into the patient samples. For example, it was shown that HNSCC of different localizations also tend to show different tumor behavior.^[27,28]^

The present analysis also identified only a weak, albeit statistically significant, correlation between SUV derived from FDG-PET and Ki 67 index in HNSCC. Therefore, SUV_max_ cannot be used as an imaging surrogate biomarker for prediction of proliferation activity in HNSCC.

Previously, some reports suggested that associations between different imaging parameters and histopathology can be influenced by tumor grade (29, 30). For example, it has been shown that the associations between SUV and ADC values derived from diffusion-weighted imaging might depend on grading in HNSCC.^[29]^ In another study, the correlations between nucleic areas and ADC values differed significantly between well and poor differentiated HNSCC tumors.^[30]^ Another study showed similar results that the associations between ADC values and immunhistochemical features, such as hypoxia-1 alpha and vascular endothelial growth factor, depend significantly on p16-status in HNSCC.^[31]^ Recently, similar results were reported also for associations between SUV values and histopathology in HNSCC.^[12]^ However, the present analysis showed that correlations between SUV and Ki 67-index were independent on tumor grade.

Furthermore, the present study identified another aspect. More aggressive, dedifferentiated tumors tend to show higher SUV values than well differentiated tumors. It is plausible that poorly differentiated, more proliferative tumors, also consume more glucose that result in a higher SUV uptake. Similar findings were reported for other tumor entities like for example breast cancer, renal cell carcinoma and pancreatic cancer.^[32–34]^ The grading system of HNSCC measures the nuclear pleomorphism, degree of keratinization, number of mitoses, pattern of invasion, and presence or absence of inflammatory infiltrates. These features might presumably be associated with glucose metabolism.

However, the reported data about relationships between SUV and tumor grade in HNSCC were also inconclusive. While some authors identified significant associations between PET parameters and tumor grades in HNSCC, other did not.^[35,36]^ For example, Fruehwald-Pallamar et al did not show significant differences of SUV between several tumor grades.^[35]^ However, Choi et al identified that poorly differentiated tumors had statistically significant higher SUV values than well and/or moderate differentiated lesions.^[36]^

The present study based on the large patient sample can confirm that SUV values increased with tumor grade, although a significant overlap between tumor groups was identified. This fact might explain why some studies could not reach statistically significance in small patient samples. Moreover, the ROC analysis only revealed a moderate ability of FDG-PET to discriminate between good/moderate and poor differentiated tumors, which limits possible translation into clinical use.

There are several limitations of the present study to address. First, it is a pooled analysis of prospective studies with possible publication bias. However, even studies with negative results were identified. Second, the PET parameters were acquired on different scanner with different protocols, which might influence the results. However, this approach also reflects the clinical routine and has a higher external validity than a single center analysis.

In conclusion, the present study identified a weak correlation between SUV values derived from FDG-PET and proliferation index Ki 67-index in HNSCC in a large patient sample. Moreover, the association is not dependent on tumor grading. Therefore, SUV_max_ cannot predict proliferation activity in HNSCC. However, SUV_max_ may aid in discrimination between well/moderate from poorly differentiated tumors.

## Author contributions

**Conceptualization:** Hans-Jonas Meyer.

**Data curation:** Hans-Jonas Meyer, Peter Gundermann.

**Formal analysis:** Hans-Jonas Meyer, Peter Gundermann.

**Methodology:** Hans-Jonas Meyer, Peter Gundermann.

**Supervision:** Alexey Surov.

**Validation:** Alexey Surov.

**Writing – original draft:** Hans-Jonas Meyer, Alexey Surov.

**Writing – review & editing:** Hans-Jonas Meyer, Alexey Surov.

## References

[1] Guizard AN Dejardin OJ Launay LC Diagnosis and management of head and neck cancers in a high-incidence area in France: a population-based study. Medicine (Baltimore) 2017;96:e7285. 28658124 10.1097/MD.0000000000007285 PMC5500046

[2] Galgano SJ Marshall RV Middlebrooks EH PET/MR imaging in head and neck cancer: current applications and future directions. Magn Reson Imaging Clin N Am 2018;26:167–78. 29128003 10.1016/j.mric.2017.08.010

[3] Minn H Lapela M Klemi PJ Prediction of survival with fluorine-18-fluoro-deoxyglucose and PET in head and neck cancer. J Nucl Med 1997;38:1907–11. 9430467

[4] Torizuka T Tanizaki Y Kanno T Prognostic value of 18F-FDG PET in patients with head and neck squamous cell cancer. AJR Am J Roentgenol 2009;192:156–60. 10.2214/AJR.08.1429 19304675

[5] Liao CT Chang JT Wang HM Pretreatment primary tumor SUVmax measured by FDG-PET and pathologic tumor depth predict for poor outcomes in patients with oral cavity squamous cell carcinoma and pathologically positive lymph nodes. Int J Radiat Oncol Biol Phys 2009;73:764–71. 18835509 10.1016/j.ijrobp.2008.05.004

[6] Kubicek GJ Champ C Fogh S FDG-PET staging and importance of lymph node SUV in head and neck cancer. Head Neck Oncol 2010;2:19. 20637102 10.1186/1758-3284-2-19 PMC2915991

[7] Jansen JF Schöder H Lee NY Tumor metabolism and perfusion in head and neck squamous cell carcinoma: pretreatment multimodality imaging with 1H magnetic resonance spectroscopy, dynamic contrast-enhanced MRI, and [18F]FDG-PET. Int J Radiat Oncol Biol Phys 2012;82:299–307. 21236594 10.1016/j.ijrobp.2010.11.022 PMC3137671

[8] Surov A Stumpp P Meyer HJ Simultaneous (18)F-FDG-PET/MRI: Associations between diffusion, glucose metabolism and histopathological parameters in patients with head and neck squamous cell carcinoma. Oral Oncol 2016;58:14–20. 27311397 10.1016/j.oraloncology.2016.04.009

[9] Surov A Meyer HJ Wienke A Can imaging parameters provide information regarding histopathology in head and neck squamous cell carcinoma? A meta-analysis. Transl Oncol 2018;11:498–503. 29510360 10.1016/j.tranon.2018.02.004 PMC5884190

[10] Higashi K Ueda Y Sakurai A Correlation of Glut-1 glucose transporter expression with [(18)F]FDG uptake in non-small cell lung cancer. Eur J Nucl Med 2000;27:1778–85. 10.1007/s002590000367 24578007

[11] Zhao ZX Lu LW Qiu J Glucose transporter-1 as an independent prognostic marker for cancer: a meta-analysis. Oncotarget 2017;9:2728–38. 29416806 10.18632/oncotarget.18964 PMC5788674

[12] Surov A Meyer HJ Höhn AK Associations Between [18F]FDG-PET and complex histopathological parameters including tumor cell count and expression of KI 67, EGFR, VEGF, HIF-1α, and p53 in head and neck squamous cell carcinoma. Mol Imaging Biol 2019;21:368–74. 29931433 10.1007/s11307-018-1223-x

[13] Rasmussen GB Vogelius IR Rasmussen JH Immunohistochemical biomarkers and FDG uptake on PET/CT in head and neck squamous cell carcinoma. Acta Oncol 2015;54:1408–15. 26256482 10.3109/0284186X.2015.1062539

[14] Denkert C Budczies J von Minckwitz G Strategies for developing Ki67 as a useful biomarker in breast cancer. Breast 2015;24 Suppl 2:S67–72. 26283598 10.1016/j.breast.2015.07.017

[15] Fischer CA Jung M Zlobec I Co-overexpression of p21 and Ki-67 in head and neck squamous cell carcinoma relative to a significantly poor prognosis. Head Neck 2011;33:267–73. 20848449 10.1002/hed.21440

[16] Szentkúti G Dános K Brauswetter D Correlations between prognosis and regional biomarker profiles in head and neck squamous cell carcinomas. Pathol Oncol Res 2015;21:643–50. 25547827 10.1007/s12253-014-9869-4

[17] Surov A Meyer HJ Wienke A Associations between apparent diffusion coefficient (ADC) and KI 67 in different tumors: a meta-analysis. Part 1: ADCmean. Oncotarget 2017;8:75434–44. 29088879 10.18632/oncotarget.20406 PMC5650434

[18] Deng SM Zhang W Zhang B Correlation between the uptake of 18F-Fluorodeoxyglucose (18F-FDG) and the expression of proliferation-associated antigen Ki-67 in cancer patients: a meta-analysis. PLoS One 2015;10:e0129028. 26038827 10.1371/journal.pone.0129028 PMC4454667

[19] Jacob R Welkoborsky HJ Mann WJ [Fluorine-18]fluorodeoxyglucose positron emission tomography, DNA ploidy and growth fraction in squamous-cell carcinomas of the head and neck. ORL J Otorhinolaryngol Relat Spec 2001;63:307–13. 11528276 10.1159/000055764

[20] Deron P Vangestel C Goethals I FDG uptake in primary squamous cell carcinoma of the head and neck. The relationship between overexpression of glucose transporters and hexokinases, tumor proliferation and apoptosis. Nuklearmedizin 2011;50:15–21. 21052609 10.3413/nukmed-0324-10-06

[21] Grönroos TJ Lehtiö K Söderström KO Hypoxia, blood flow and metabolism in squamous-cell carcinoma of the head and neck: correlations between multiple immunohistochemical parameters and PET. BMC Cancer 2014;14:876. 25421331 10.1186/1471-2407-14-876 PMC4251851

[22] Hoshikawa H Nishiyama Y Kishino T Comparison of FLT-PET and FDG-PET for visualization of head and neck squamous cell cancers. Mol Imaging Biol 2011;13:172–7. 20464518 10.1007/s11307-010-0331-z

[23] Kim M Achmad A Higuchi T Effects of intratumoral inflammatory process on 18F-FDG uptake: pathologic and comparative study with 18F-fluoro-α-methyltyrosine PET/CT in oral squamous cell carcinoma. J Nucl Med 2015;56:16–21. 25476535 10.2967/jnumed.114.144014

[24] Kitagawa Y Sano K Nishizawa S FDG-PET for prediction of tumour aggressiveness and response to intra-arterial chemotherapy and radiotherapy in head and neck cancer. Eur J Nucl Med Mol Imaging 2003;30:63–71. 12483411 10.1007/s00259-002-0978-z

[25] Linecker A Kermer C Sulzbacher I Uptake of (18)F-FLT and (18)F-FDG in primary head and neck cancer correlates with survival. Nuklearmedizin 2008;47:80–5. 18392317 10.3413/nukmed-0092

[26] Miyashita G Higuchi T Oriuchi N 18F-FAMT uptake correlates with tumor proliferative activity in oral squamous cell carcinoma: comparative study with 18F-FDG PET and immunohistochemistry. Ann Nucl Med 2010;24:579–84. 20652456 10.1007/s12149-010-0398-2

[27] Tamás L Szentkúti G Eros M Differential biomarker expression in head and neck cancer correlates with anatomical localization. Pathol Oncol Res 2011;17:721–7. 21487776 10.1007/s12253-011-9376-9

[28] Takes RP Baatenburg de Jong RJ Schuuring E Differences in expression of oncogenes and tumor suppressor genes in different sites of head and neck squamous cell. Anticancer Res 1998;18:4793–800. 9891559

[29] Leifels L Purz S Stumpp P Associations between 18F-FDG-PET, DWI, and DCE parameters in patients with head and neck squamous cell carcinoma depend on tumor grading. Contrast Media Mol Imaging 2017;5369625. 29114177 10.1155/2017/5369625 PMC5671689

[30] Surov A Meyer HJ Leifels L Histogram analysis parameters of dynamic contrast-enhanced magnetic resonance imaging can predict histopathological findings including proliferation potential, cellularity, and nucleic areas in head and neck squamous cell carcinoma. Oncotarget 2018;9:21070–7. 29765520 10.18632/oncotarget.24920 PMC5940412

[31] Meyer HJ Leifels L Hamerla G ADC-histogram analysis in head and neck squamous cell carcinoma. Associations with different histopathological features including expression of EGFR, VEGF, HIF-1α, Her 2 and p53 A preliminary study. Magn Reson Imaging 2018;54:214–7. 30189236 10.1016/j.mri.2018.07.013

[32] García Vicente AM Castrejón ÁS Relea Calatayud F 18F-FDG retention index and biologic prognostic parameters in breast cancer. Clin Nucl Med 2012;37:460–6. 22475895 10.1097/RLU.0b013e31823926c9

[33] Noda Y Kanematsu M Goshima S 18-F fluorodeoxyglucose uptake in positron emission tomography as a pathological grade predictor for renal clear cell carcinomas. Eur Radiol 2015;25:3009–16. 25854217 10.1007/s00330-015-3687-2

[34] Ahn SJ Park MS Lee JD Correlation between 18F-fluorodeoxyglucose positron emission tomography and pathologic differentiation in pancreatic cancer. Ann Nucl Med 2014;28:430–5. 24623151 10.1007/s12149-014-0833-x

[35] Fruehwald-Pallamar J Czerny C Mayerhoefer ME Functional imaging in head and neck squamous cell carcinoma: correlation of PET/CT and diffusion-weighted imaging at 3 Tesla. Eur J Nucl Med Mol Imaging 2011;38:1009–19. 21465255 10.1007/s00259-010-1718-4

[36] Choi SH Paeng JC Sohn CH Correlation of 18F-FDG uptake with apparent diffusion coefficient ratio measured on standard and high b value diffusion MRI in head and neck cancer. J Nucl Med 2011;52:1056–62. 21680692 10.2967/jnumed.111.089334

